# New Optical Imaging Reporter-labeled Anaplastic Thyroid Cancer-Derived Extracellular Vesicles as a Platform for *In Vivo* Tumor Targeting in a Mouse Model

**DOI:** 10.1038/s41598-018-31998-y

**Published:** 2018-09-10

**Authors:** Prakash Gangadaran, Xiu Juan Li, Senthil kumar Kalimuthu, Oh Ji Min, Chae Moon Hong, Ramya Lakshmi Rajendran, Ho Won Lee, Liya Zhu, Se Hwan Baek, Shin Young Jeong, Sang-Woo Lee, Jaetae Lee, Byeong-Cheol Ahn

**Affiliations:** 1 Department of Nuclear Medicine, School of Medicine, Kyungpook National University, Daegu, Republic of Korea; 2 Department of Nuclear Medicine, Kyungpook National University Hospital, Daegu, Republic of Korea; 3 Department of Radiology, Taian City Central Hospital, Taian, People’s Republic of China

## Abstract

Extracellular vesicles (EVs), originating from multivesicular bodies by invagination of the endosomal membrane, are communication channels between distant cells. They are natural carriers of exogeneous cellular materials and have been exploited as drug delivery carriers in various diseases. Here, we found that tumor cell-derived EVs can be used as efficient targets in tumors by monitoring with an optical reporter system. Anaplastic thyroid cancer (CAL62) cell-derived EVs with *Renilla* luciferase (Rluc) were used to target CAL62 tumors in a mouse model. Optical imaging revealed that cancer cell-derived EVs (EV-CAL62/Rluc) targeted the original tumor (CAL62) in mice within 30 min after systemic injection. Furthermore, fluorescence imaging revealed that EV-CAL62/Rluc were internalized into CAL62 tumors in the mice. *Ex vivo* Optical imaging further confirmed the *in vivo* finding. Here, we successfully monitored the tumor targeting ability of tumor cell-derived EVs by optical imaging. Based on these results, tumor cell-derived EVs are highly effective natural carriers for drug delivery for cancer therapies.

## Introduction

Naturally produced biological nanoparticles are known as extracellular vesicles (EVs). EVs are released from cells into the extracellular space and found in various body fluids such as the blood, urine, and central nervous system fluids^1–3^. EVs are classified into exosomes and microvesicles. Exosomes (50–200 nm) are membrane vesicles released by multi-vesicular bodies. Microvesicles (50–1000 nm) are released from the cell membrane via a budding process in the cell and are larger than exosomes^4,5^. EVs are capable of carrying vary biological materials such as lipids, mRNA, miRNA, proteins, and extrachromosomal DNA^3,6–9^. Cancer cells produce and secrete larger numbers of EVs compared to normal cells^10^. Tumor-derived EVs are information carriers that convey molecular and genetic messages from tumor cells to normal or other abnormal cells residing at close or distant sites^11^.

EVs are a novel class of intercellular signal mediators that are involved in a number of different physiological and pathological processes^11,12^. Previous studies suggested that the contact between EVs and recipient cells occurs through receptor-ligand binding^13–15^. Another study showed that primary melanoma exosomes can be preferentially delivered to metastatic melanoma tumor cells^16^. A recent study used cancer-derived exosomes as a useful delivery vehicle with low immunogenicity for efficient CRISPR/Cas9-mediated genome editing in cancer cells. Particularly, cancer-derived exosomes showed preferential uptake to into cancer cells compared to epithelial cell-derived exosomes^17^. Tumor targeting and selective drug delivery using cancer-derived EVs has been proposed because of their specific expression of tetraspanins, which preferentially interact with certain ligands^18^. The precise mechanisms of these interactions are not clearly understood. Few studies have demonstrated that tumor-derived EVs can target a parental tumor *in vitro* and *in vivo*^17^, and further studies are necessary to assess this phenomenon *in vivo*. These characteristics of tumor-derived EVs can be used to target the tumor and in drug delivery vehicles. EVs are excellent endogenous nanocarriers for exogeneous drug delivery systems. In recent years, several studies showed that drug-loaded EVs improved disease conditions^19^, including studies performed using different cancer models^20–23^. One way of reducing side effects is to target the delivery of an anti-cancer drug to the tumor.

Recent studies showed that EVs can be visualized in *in vivo* animal models by using a lipophilic dye^24^, radionuclides^25,26^, magnetic particles^27,28^ and bioluminescence reporter system^29^. The labeling process with lipophilic dyes is simple and the labeling is suitable for real-time monitoring of EVs, but lipophilic dyes promotes clumping of EVs^30^; significant EV damage^29^; non-specific signals from dye remains in tissues^5,29^. Nuclear imaging could be a good candidate for tracking EVs in both preclinical and clinical studies, a limitation of this technology is the possibility of altering EV characteristics by the labeling procedure^5^. In recent studies, EVs were loaded with MRI contrasts and visualized using MRI^27,28,31^. A large amount of iron oxide-loaded EVs are needed as low sensitivity of MRI technology^5,28^. In preclinical studies bioluminescent imaging (BLI) has an extremely high signal-to-noise ratio, low auto-luminescence in mammalian tissue; low background emission compared to fluorescent-based imaging^32,33^.

Noninvasive or *in vivo* bioluminescent imaging is particularly advantageous for studying various live cells in small animals^32,34,35^. We recently developed a highly sensitive *in vivo* visualization method for EVs by employing a new BLI reporter (*Renilla luciferase*) system^33^. Studies involving real-time visualization of tumor-derived EVs as tumor-targeting agents are urgently needed. The present study was performed to determine whether EVs can preferentially target their parent cell, which may be useful for tumor targeting and drug delivery. Here, we tested a newly developed bioluminescent EV reporter system in an *in vivo* animal model to monitor the targeting ability of thyroid cancer-derived EVs to original tumors.

## Results

### Generation of Stable Cell Lines Expressing Luciferase Reporter Genes

**Figure 1:**
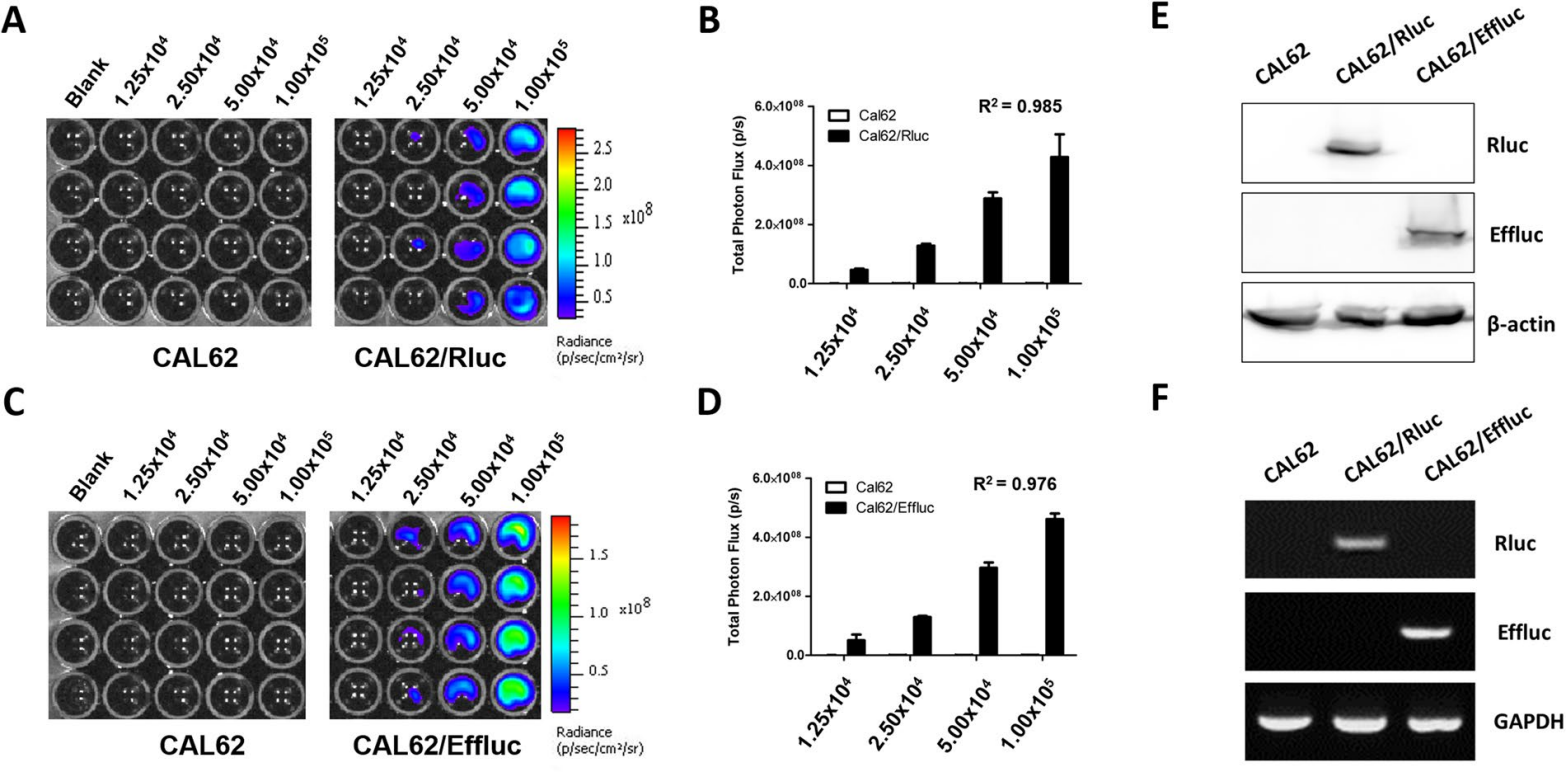
Generation of stable reporter gene expression in a cancer cell line. (**A**) Representative bioluminescent imaging of the *in vitro* luciferase assay in CAL62 and CAL-62/Rluc cells. (**B**) *In vitro* luciferase assay in CAL62 and CAL62/Rluc cells. Data are expressed as the mean ± standard deviation (SD). (**C**) Representative bioluminescent imaging of the *in vitro* luciferase assay in CAL62 and CAL62/Effluc cells. (**D**) *In vitro* luciferase assay in CAL62 and CAL62/Effluc cells. Data are expressed as the mean ± standard deviation (SD). (**E**) Western blot analysis of the Rluc and Effluc proteins in CAL62, CAL62/Rluc, and CAL62/Effluc cells; β-actin was used as an internal control. (**F**) RT-PCR analysis to determine the expression of the Rluc and Effluc genes in CAL62, CAL62/Rluc, and CAL62/Effluc cells; GAPDH served as an internal control.

Anaplastic thyroid cancer cells (CAL62) transfected with retrovirus containing the Rluc gene or effluc gene were used to generate cells expressing a reporter gene. CAL62 cells transduced with the Rluc gene were named as CAL62/Rluc and those transduced with the Effluc gene were named as CAL62/Effluc. Successful insertion of the Rluc or effluc gene into CAL62 cells was confirmed by BLI, as shown in Fig. 1A–D. As the BLI signal in CAL62/Rluc and CAL62/Effluc cells increased, there was increase in BLI signal in dose-dependent manner. (Cal62/Rluc: R^2^ = 0.985; Cal62/Effluc: R^2^ = 0.976) and no signals were observed in the parental CAL62 cells. In addition, successful transduction of the Rluc or Effluc gene into cells was confirmed by RT-PCR and western blotting (Figs 1E,F and S1A,B). Taken together, these results indicate that Rluc and Effluc were stably expressed in CAL62 cells. CAL62/Rluc cells were used for the isolation of EVs, and CAL62/Effluc cells were used to prepare a subcutaneous tumor mouse model.

### Characterization of EV-CAL62/Rluc

**Figure 2:**
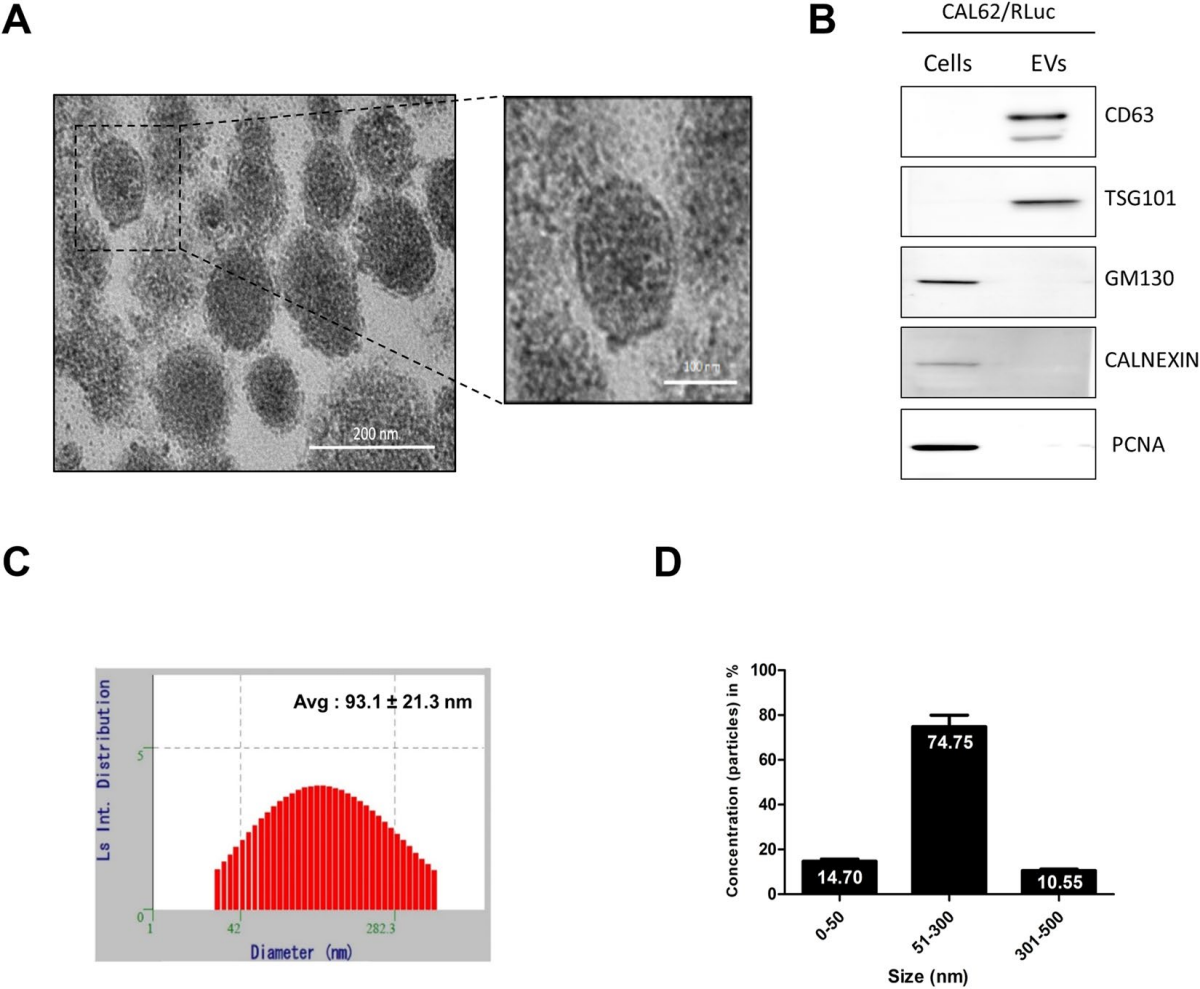
Characterization of EVs. (**A**) Examination of EVs from CAL62/Rluc cells using electron microscopy Scale bar: 100, 200 nm. (**B**) Western blotting analysis of EVs. CD63 and TSG101 (EV marker proteins) GM130, CALNEXIN and PCNA (cell marker proteins) were detected by specific antibodies. (**C**,**D**) EV size and concentrations were determined by DLS (Avg. 93.1 ± 21.3 nm). (**D**) Distribution of EVs population in the isolated EVs.

We isolated EVs (EV-CAL62/Rluc) from conditioned medium obtained from CAL62/Rluc cells by ultracentrifugation as described previously^33^. EV-CAL62/Rluc was examined by TEM, which revealed that the EVs were 100–300-nm round shape vesicles with an intact membrane structure, which are recognized characteristics of EVs (Fig. 2A). Further, western blotting analysis confirmed the enrichment of EVs-biomarkers such as tetraspanins protein cluster of differentiation 63 (CD63) and tumor susceptibility gene 101 (TSG101) in the EVs. Furthermore, absence of Golgin subfamily A member 2 (GM130), the endoplasmic reticulum protein (Calnexin) and proliferating cell nuclear antigen (PCNA) was confirmed and these proteins were present in cells (Figs 2B and S2A). In addition, the average size and range concentration of EVs were analyzed by ELS, which revealed that average size of EVs was 93.1 ± 21.3 nm and approximately 75% of EVs were 50–300 nm (Fig. 2C,D). Together, these results confirm the characterization and purification of isolated EVs.

### Renilla Luciferase Activity of EV-CAL62/Rluc

**Figure 3:**
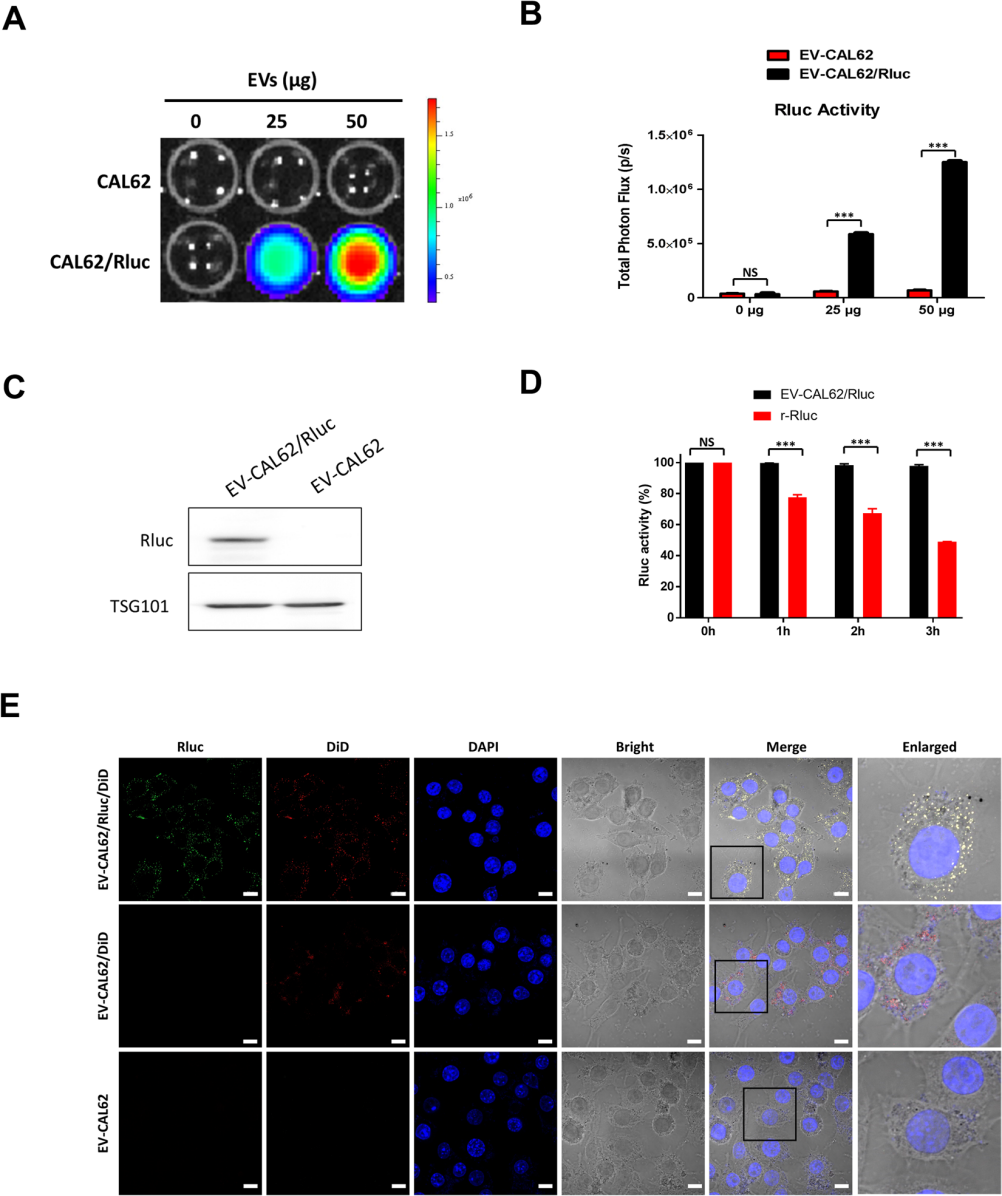
EV-CAL-62/Rluc showed EV-specific Rluc activity and serum stability *in vitro*. (**A**) Representative bioluminescent imaging of an *in vitro* luciferase assay in EVs from CAL-62 and CAL-62/Rluc cells. (**B**) Quantitative *in vitro* luciferase data in EVs are expressed as the mean ± SD. (**C**) Western blot analysis of the Rluc protein in EVs from CAL-62, CAL-62/Rluc cells, detected by means of Rluc-specific antibodies. TSG served as loading control. (**D**) Stability of Rluc in EV-CAL62/Rluc and recombinant Rluc protein in serum. Time course of stability of Rluc at 37 °C in 20% FBS/PBS buffer. (**E**) Representative confocal images of Rluc (green) and DiD (red) in EV-CAL62/Rluc/DiD or EV-CAL62/DiD or EV-CAL62 in CAL62 cells. Scale bars: 10 μm.

To confirm the presence of Rluc protein in CAL62 cell-derived EVs, BLI was performed, which revealed that Rluc levels and BLI signals increased in a dose-dependent manner (Fig. 3A,B; P < 0.001). The western blotting analysis further confirmed the presence of Rluc protein in EV-CAL62/Rluc and not in EV-CAL62 (Figs 3C and S2B).

### Rluc in EV-CAL62/Rluc Showed High Stability in the Serum

The Rluc stability of EV-CAL62/Rluc and recombinant Rluc (r-Rluc) in serum was tested. Rluc activity was decreased marginally EV-CAL62/Rluc, the percentage of serum stability of Rluc in EV-CAL62/Rluc was approximately 98% (1 h), 98% (2 h) and 97% (3 h) but r-Rluc serum stability was significantly decreased (P < 0.001) compare to EV-CAL62/Rluc and it was approximately 77% (1 h), 58% (2 h) and 47% (3 h) (Fig. 3D).

### Immunofluorescent (IF) Imaging Confirms the Presence of Rluc in EV-CAL62/Rluc

EV-CAL62/Rluc or EV-CAL62 were labeled with NIR dye-DiD (a fluorescent lipophilic tracer) and then incubated with CAL62 cells. After 2-hour, IF was performed with Rluc antibody. IF results revealed the presence of Rluc signals in the EV-CAL62/Rluc/DiD incubated CAL62 cells and no Rluc signals present in EV-CAL62 /DiD incubated CAL62 cells. Further, Rluc signals were co-localized with DiD signals in EV-CAL62/Rluc/DiD incubated CAL62 cells. Whereas only DiD signals were observed in EV-CAL62/DiD incubated CAL62 cells (Fig. 3E).

### EV-CAL62 are internalized by CAL62 and CAL62/Effluc Cells

**Figure 4:**
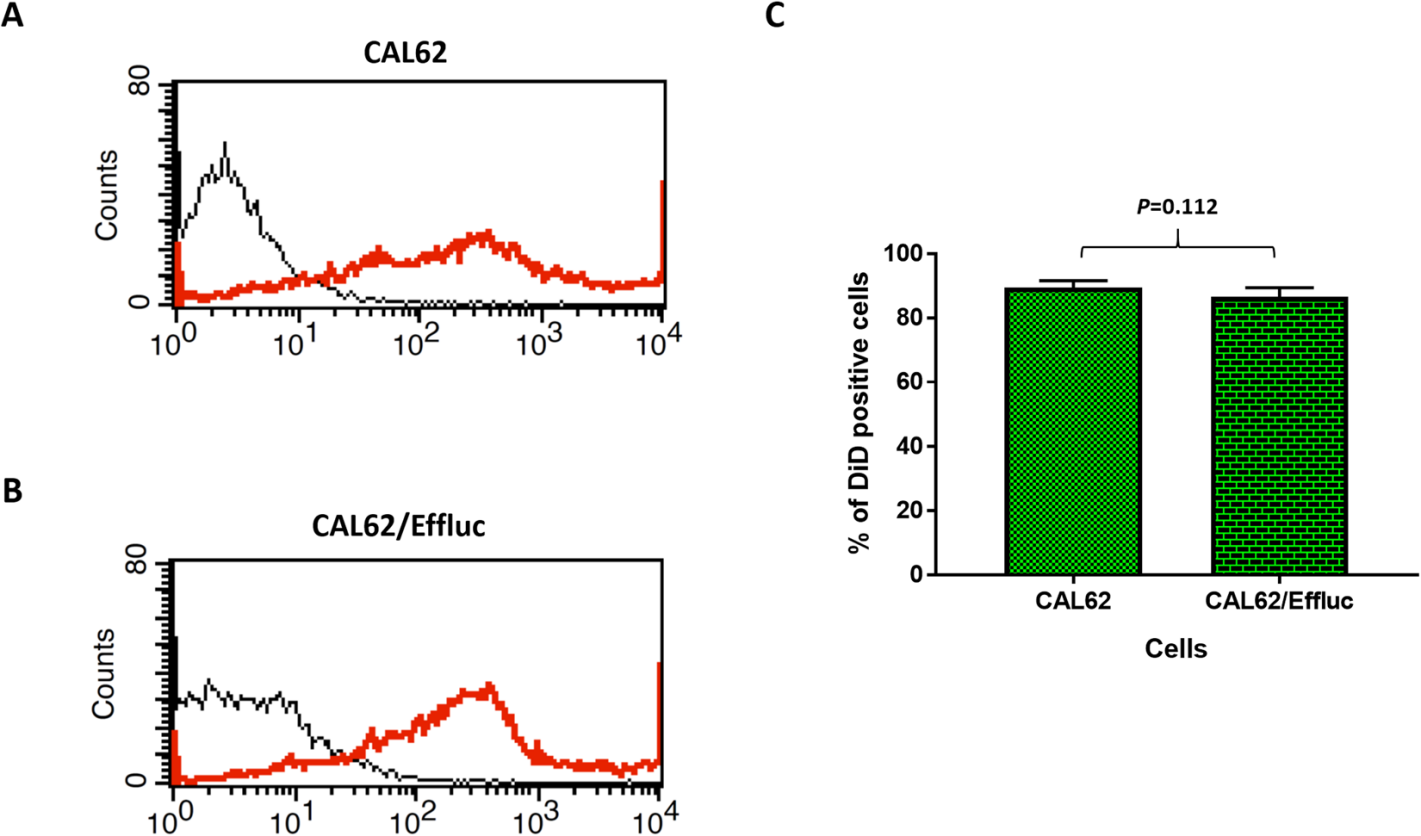
Internalization of EVs into cancer cells. (**A**,**B**) Representative flowcytometry images of CAL62 or CAL62/Effluc cells incubated with unlabeled EV-CAL62/Rluc or DiD-labeled EV-CAL62/Rluc. (**B**) Quantification of DiD positive cells from flowcytometry of CAL62 or CAL62/Effluc cells treated with unlabeled EV-CAL62/Rluc or DiD-labeled EV-CAL62/Rluc was represented in bar graph (p = 0.112).

EV-based drug delivery to cells is possible by internalization of EVs into targeted cells. Therefore, we measured the internalization of EV-CAL62/Rluc into recipient parent CAL62 and CAL62/Effluc cancer cells. EVs were purified and labeled with the NIR dye-DiD. Confocal laser microscopy, performed 1 h after the incubation of EVs, revealed the effective internalization of EV-CAL62/Rluc into CAL62 and CAL62/Effluc cells (Supplementary Fig. 3A). Total number of DiD (EV-CAL62/Rluc/DiD) positive cells were not significantly (*P* = 0.349) changed between CAL62 (58.7) and CAL62/Effluc (60.5) cells (Supplementary Fig. 3B). Further, we also confirmed the internalization of EVs using flow cytometry. Results revealed that EV-CAL62/Rluc were effectively internalized into CAL62 and CAL62/Effluc cells (Fig. 4A,B). Total number of DiD (EV-CAL62/Rluc/DiD) positive cells were not significantly (*P* = 0.112) different between CAL62 (89.5%) and CAL62/Effluc (86.6%) cells (Fig. 4C). These results indicated that EV-CAL62/Rluc actively internalized irrespective of effluc gene expression in CAL62 cells.

### *In vivo* Tumor Targeting Monitored by Bioluminescent Imaging

**Figure 5:**
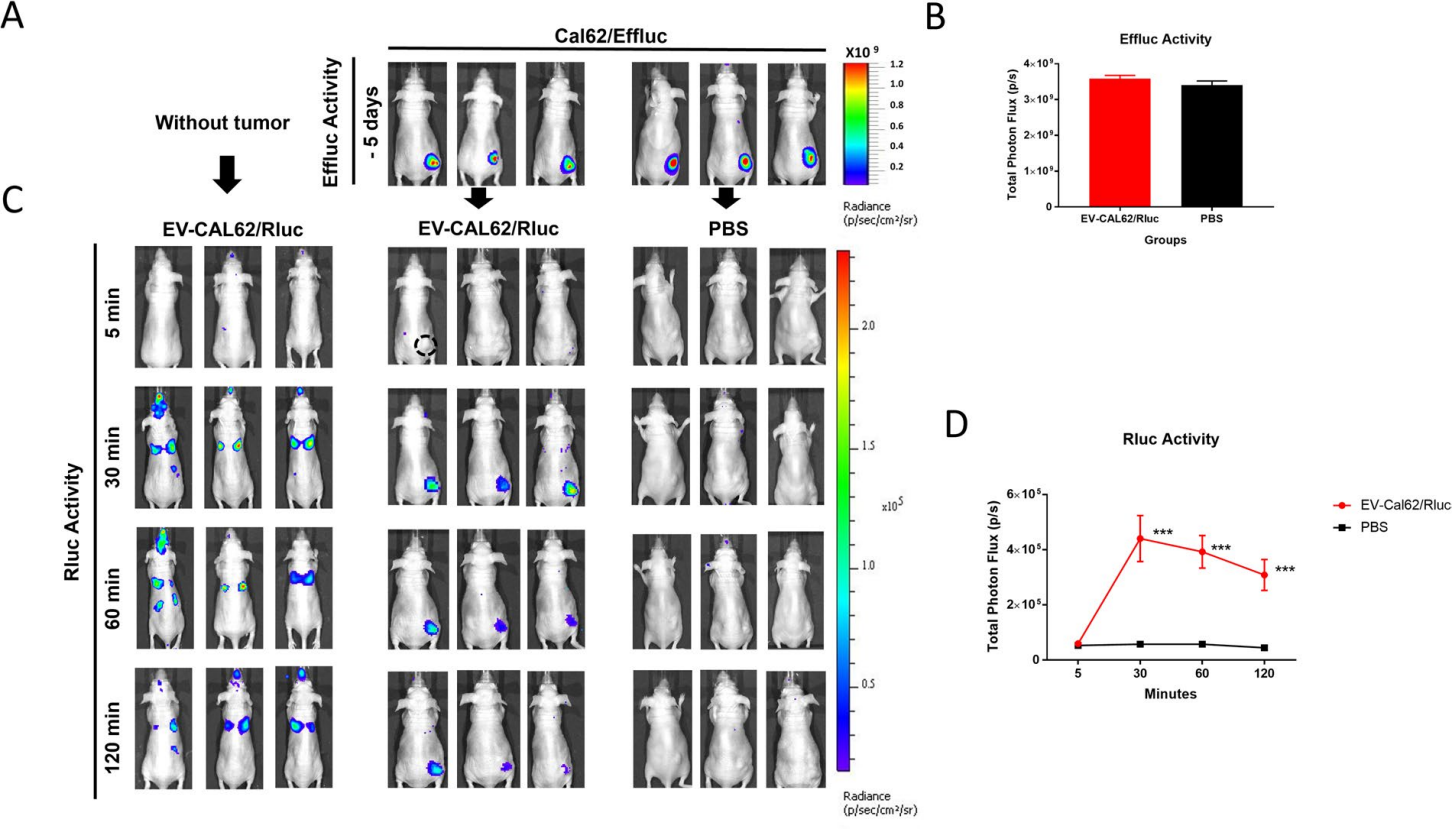
*In vivo* visualizing and monitoring tumor-derived EVs targeting the same tumor. (**A**) Image of Effluc activity of CAL62/Effluc nude mice at six weeks after subcutaneous injection of CAL62/Effluc cells. (**B**) Quantification of tumor BLI signals from derived EV-CAL62/Rluc (n = 3) or PBS (n = 3) mouse groups.(**C**) *In vivo* imaging of EV-CAL62/Rluc intravenously administered to naïve or CAL62/Effluc tumor-bearing mice. (**D**) Quantification of Rluc (EV-CAL62/Rluc) signals in the tumor region of mice mentioned in 3C.

CAL62/Effluc cells were subcutaneously injected into mice and the mice were randomly divided into two groups (EV-CAL62/Rluc and PBS) at the 6^th^ week. The tumor was assessed by BLI after intraperitoneal D-luciferin injection 5 days prior to EV administration (Fig. 5A,B). We isolated EVs (EV-CAL62/Rluc) from conditioned medium obtained from CAL62/Rluc cells by ultracentrifugation as described previously^33^. We intravenously administered 50 µg of EV-CAL62/Rluc to mice with or without tumor xenograft, while control mice were administered PBS. Coelenterazine was intravenously injected at 5, 30, 60, and 120 min after EV-Cal62/Rluc (50 µg) or PBS injection and BLI was obtained using an IVIS Lumina III.

BLI showed that no signals were visualized at 5 min post-injection all the three groups and EV-CAL-62/Rluc was visualized in the region of lungs, liver and spleen of mice and in the tumor of CAL62/Effluc tumor bearing mice within 30 min after injection (Fig. 5C,D); quantitative analysis revealed that significant (*P* < 0.001) BLI signals were observed in the tumor (CAL62/Effluc) region at 30 min and No signals were visualized at the tumor site (CAL62/Effluc) in PBS-injected mice at 30 min by imaging (Fig. 5C,D). Furthermore, BLI imaging at 60 and 120 min revealed that EV-CAL62/Rluc remained in the region of lung in mice without tumor xenograft and in the tumor of CAL62/Effluc tumor bearing mice (Fig. 5C,D). Quantitative analysis revealed significantly (*P* < 0.001) higher BLI signals in the tumor region (CAL62/Effluc) at 60 and 120 min in EV-CAL62/Rluc-injected CAL62/Effluc tumor bearing mice compared to those in control PBS-injected CAL62/Effluc tumor bearing mice (Fig. 5D). Taken together, these results revealed that EV-CAL62/Rluc are predominantly accumulated in lung of mice without tumor xenograft and accumulated in the tumor of tumor bearing mice.

### *Ex Vivo* Bioluminescent Imaging of EV-CAL62/Rluc Targeting Tumors and Subcellular Visualization EV-CAL62/Rluc by Fluorescent Imaging

**Figure 6:**
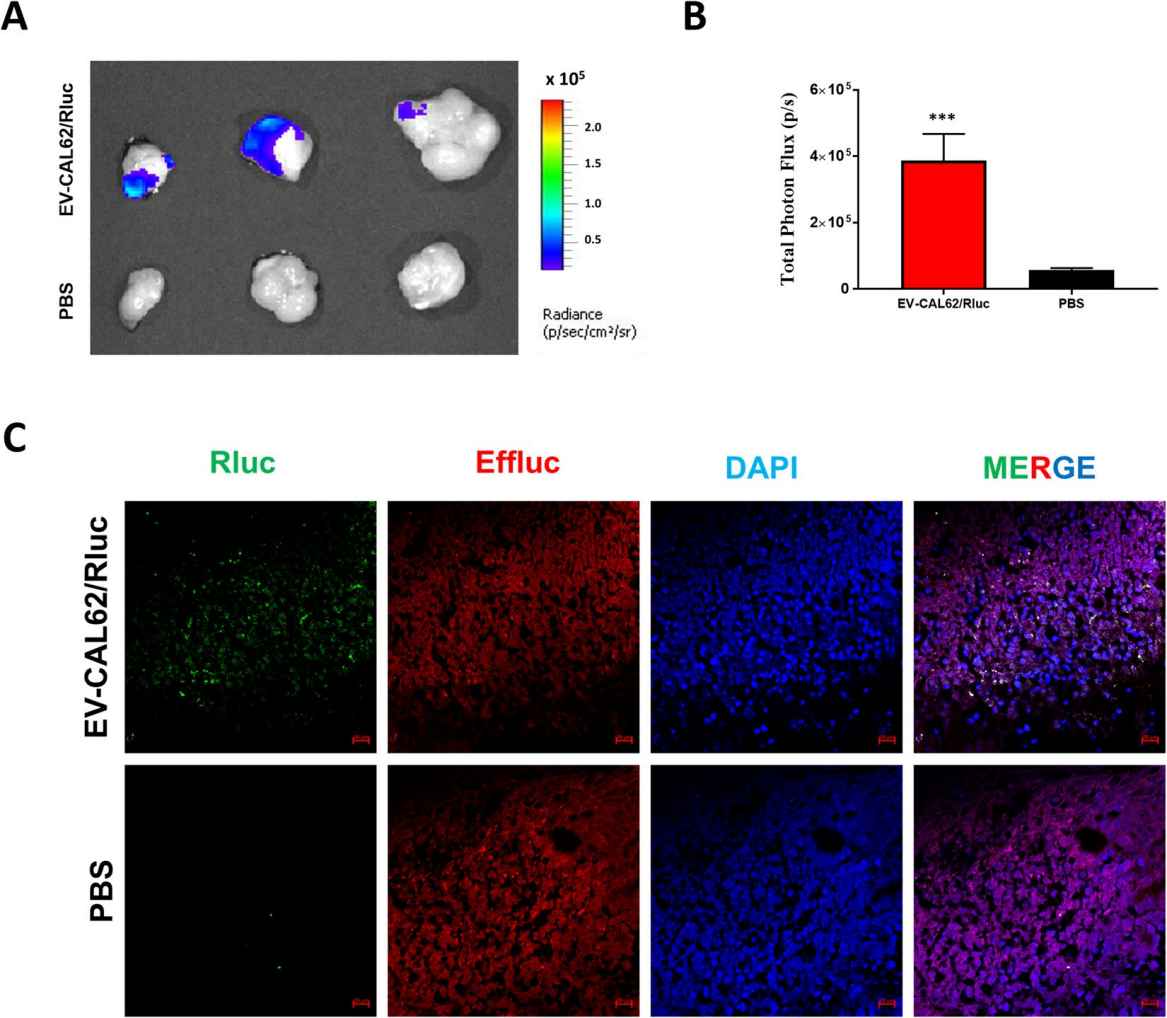
*Ex vivo* and Subcellular visualization of EV-CAL62/Rluc in tumors. (**A**) Representative *ex vivo* bioluminescent imaging of EV-CAL62/Rluc excised tumor from EV-CAL62/Rluc or PBS intravenously administered to CAL62/Effluc tumor-bearing mice (n = 3). (**B**) Quantification of EV-CAL62/Rluc signal from the tumor above mentioned in (**A**); the values are expressed as mean ± SD. (**C**) Representative confocal images of Rluc in harvested tumors (CAL62/Effluc) from mice described in Fig. 3C after 120 min. Scale bars: 50 μm.

*Ex vivo* bioluminescent imaging of excised tumor showed bioluminescent signals after incubated with Coelenterazine in EV-CAL62/Rluc injected mice and no signals were observed in PBS control (Fig. 6A). These results further confirmed the *in vivo* imaging of EV-CAL62/Rluc targeting CAL62/Effluc Tumor. The quantification of bioluminescent imaging showed significant signals (P < 0.001) in EV-CAL62/Rluc injected mice compare to PBS control (Fig. 6B). Tumors were sectioned and subjected to immunofluorescent staining with Rluc- and Effluc-specific antibodies, which revealed that EV-CAL-62/Rluc localized to the CAL62/Effluc tumor. No Rluc signals were observed in PBS control tumors (CAL62/Effluc) (Fig. 6C).

## Discussion

EVs are naturally occurring carriers that can target tumors and deliver a wide range of endogenous and exogeneous and small molecules, anti-cancer drugs, and nucleic acids^20,36–38^. Thus, using EVs as drug delivery vehicles shows more potential compared to other nanoparticles that induce high immunogenicity *in vivo* and are easily eliminated by the immune system^17,39^. Tumor-derived EVs affect tumors in various manners, including effects on the epithelial to mesenchymal transition in nasopharyngeal carcinoma^40^, converting mesenchymal stem cells into cancer-associated fibroblasts^41^, transporting paracrine signaling factors and mRNA to induce angiogenesis in the metastatic microenvironment^42^, and facilitating organotropic metastatic growth^43,44^, among others. EV proteins and miRNA can be used as biomarkers for the early detection of tumors^45,46^. Tumor-derived EVs can be used for cell-free anti-tumor vaccination to induce T-cell activation and T-cell dependent immune responses against tumor cells *in vivo*^47^. They also have antitumor immunogenicity even without expressing MHC-I molecules, which can provide antigens against various cancer types^48^.

*In vivo* visualization and monitoring the EVs is an animal model can provide a foundation for developing EV-based therapies. Here, we tested the targeting ability of tumor-derived EVs to its parental tumor *in vivo* in a mouse model by using a newly developed reporter gene-based optical imaging system for *in vivo* EV monitoring, which may be useful as a monitoring platform for EV-based delivery vehicles. We used anaplastic thyroid cancer cells (CAL62) to test our hypotheses that CAL62 cancer cell-derived EVs can target CAL62 tumors in an *in vivo* animal model. We successfully inserted an optical reporter gene (Rluc) in CAL62 cells and isolated EVs with the optical reporter protein (Rluc). We also separately transduced CAL62 cells with the Effluc gene to prepare a subcutaneous tumor mouse model.

First, we isolated EVs from the CAL62/Rluc cell culture media using a conventional ultracentrifugation method. The round morphology, size, positive or negative and Rluc Protein in EV-CAL62/Rluc which is consistent with previous study^33^. Rluc in the EV-CAL62/Rluc showed a strong stability compare to r-Rluc under serum condition and consistent with other study^33^. Furthermore, Immunofluorescent assay confirmed the Rluc present in the EVs compartment. Immunofluorescent and flowcytometry analysis confirmed the EV-CAL62/Rluc are able to internalize into the same cancer cells more effectively regardless of Effluc gene expression in the cancer cells, This results support the hypothesis that cancer cell-derived EVs harbor a specific tropism, based on their cellular origin^17^, which can be exploited to deliver the drugs into cancer cells.

Furthermore, we used a newly developed optical reporter system to track EVs *in vivo*; the results suggested that most EVs accumulated in the tumor, while previous studies showed that a portion or more than half of tumor-derived EVs accumulated in the same tumor in *in vivo*^14,17^. Our previous work showed that labeling EVs with lipophilic dye affected their biodistribution *in vivo*^33^, as EV targeting occurs through integrins present in the EV membrane^49^, which can be blocked by dye labeling on the EV surface membrane and can lead to less accumulation at the tumor *in vivo*. Further, we visualized the subcellular distribution of EV-CAL-62/Rluc in excised tumors by immunofluorescence. Our *ex vivo* results further confirmed the *in vivo* finding and further results showed that most EVs were co-localized with CAL-62/Effluc cells, and previous studies found that cancer cells readily internalize EVs to a greater extent than normal cells^50^.

We confirmed that EVs derived from the tumor targeted the same tumor when injected intravenously into an *in vivo* mouse model. BLI imaging clearly showed that intravenously injected EV-CAL62/Rluc accumulated at the tumor region of mice at 30 min after injection but EV-CAL62/Rluc injected into nude mice without tumor xenograft by the intravenous route accumulated predominantly in the lung and liver, which is consistent with previous study^33^. Similar to our results (EV-CAL62 targeting CAL62 cells *in vivo*), a recent study evaluated exosomes derived from tumor cells (SKOV3) injected intravenously into the tail veins of SKOV3 xenograft mice; optical imaging revealed that Cy5.5-labeled SKOV3-Exo was significantly accumulated in tumor sites *in vivo*^17^. Other studies suggested that tumor-derived EVs are more highly associated with cancer cells than with normal cells, and an acidic pH may be one factor affecting tumor exosome trafficking to the tumor^16^. Another study suggested that tumor-derived EVs have unique protein and lipids compartments resembling those of cells from which they are derived and may interact very uniquely with the same tumor cells^50^. In addition, more SKOV3-derived exosomes were internalized than HEK293-derived exosomes in SKOV3 tumors^17^. Based on these data, tumor-derived EVs are an attractive therapeutic cargo carrier for targeted drug delivery systems. Furthermore, tumor derived EVs can be administered via intracardiac or intraperitoneal routes to enhance tumor targeting ability in the future.

Recently, EVs were considered as new biological drug delivery vehicles. EVs are small and have a negative zeta potential to ensure long circulation^51^ and they can escape degradation^52^ and can evade the immune system^53^. EVs can carry drugs, DNA, mRNA, miRNA, and proteins^54^. In this study, Rluc proteins inside the EVs were successfully delivered to the target tissue. A recent study of cancer-derived EVs suggested that they can promote cancer progression and form a pre-metastatic niche^54^; advancements in the engineering of EVs may reduce these effects of cancer-derived EVs. Furthermore, drugs can be effectively delivered to the tumor area using cancer-derived EVs to destroy cancer cells. Cancer-derived EVs are produced in greater numbers than EVs in normal cells, reducing the effort needed to collect EVs.

## Conclusion

We successfully visualized EVs derived from CAL-62 targets CAL-62 tumors *in vivo* using an optical imaging system. These cancer-derived EVs show potential as candidates for drug delivery systems.

## Materials and Methods

### Cell Culture and Transduction

An anaplastic thyroid cancer cell line (ATC), CAL-62, was cultured in Dulbecco’s modified Eagle medium (DMEM, Gibco, Grand Island, NY, USA) supplemented with 10% fetal calf serum (Hyclone, Logan, UT, USA) and 1% antibiotics (Gibco) and incubated at 37 °C in a 5% CO_2_ atmosphere. CAL-62 cells were transduced with a lentivirus expressing Rluc with puromycin genes under control of the CMV promoter (Genecopoeia, Rockville, MD, USA)^33^. CAL-62 cells were retrovirally transduced to express both effluc and thy1.1. Thy1.1-positive cells were sorted using CD90.1 micro-beads (Miltenyi Biotec, Bergisch Gladbach, Germany)^55^.

### Luciferase Activity Assays

CAL-62 cells, CAL-62/Rluc and CAL-62/Effluc (1.25 × 10^4^, 2.5 × 10^4^, 5 × 10^4^, and 10^5^ cells/well) were plated into white and clear-bottom 96-well plates containing serum-free DMEM. Twenty-four hours later, 5 µl of coelenterazine/100 µl media (15 µg/ml final concentration) for Rluc or 2 µl D-luciferin/100 µl media (15 µg/ml final concentration) for Effluc substrate (Caliper, PerkinElmer, Waltham, MA, USA) was added to each well. Fluc emits photons in a reaction that requires ATP, Mg^2+^, oxygen, and D-luciferin^56^. In the presence of oxygen, Rluc catalyzes the non-ATP-dependent oxidation of coelenterazine to generate a luminescent signal^57^. Rluc or Effluc activity was determined by BLI using the IVIS Lumina III instrument (*In Vivo* Imaging System, IVIS Lumina III, PerkinElmer).

### RT-PCR Analysis

CAL-62, CAL-62/Rluc and CAL-62/Effluc cells were lysed using Trizol solution (Invitrogen, Carlsbad, CA, USA), and total RNA was extracted according to the manufacturer’s instructions. Reverse transcription was performed as previously described^33^ using a High-Capacity cDNA Reverse Transcription Kit (Thermo Fisher Scientific, Inc., Waltham, MA, USA). After denaturing the samples for 2 min at 94 °C, 40 cycles for 20 s at 94 °C, 10 s at 57 °C, and 30 s at 72 °C were conducted with an additional 5 min at 72 °C. DNA polymerase (Takara, Shiga, Japan) and a Takara PCR system were used. Primers sequences were as follows: Rluc; forward: 5′-TATGATTCCGAGAAGCACGC-3′; reverse: 5′-TGATCCAGGAGGCGATATGA-3′; Fluc; forward: 5′-GCACAAGGCCATGAAGAGAT-3′; reverse: 5′-CTTCTTGCTCACGAACACCA-3′; GAPDH; forward: 5′-AGTGATGGCATGGACTGTGG-3′; reverse: 5′-GTCAAGGCTGAGAACGGGAA-3′. Samples were separated by electrophoresis on an ethidium bromide-stained agarose gel. Gels were imaged on a UV trans-illuminator using a UVP Gel-Doc-IT imaging system.

### Western Blot Analysis

Western blotting was performed as described previously^33^. Whole-cell or EV lysates were prepared in RIPA buffer (Thermo Fisher Scientific) with protease inhibitor cocktail (Thermo Fisher Scientific). An equal amount of protein (20 µg per well) was loaded and separated by 10% SDS–PAGE. The proteins were transferred from the gel to a polyvinylidene fluoride membrane (Millipore, Billerica, MA, USA) and probed with the primary antibody and then with a secondary antibody conjugated to horseradish peroxidase (HRP). The following primary antibodies were used: CD63, TSG101, GM130, Calnexin, Rluc (Abcam, Cambridge, UK; Dilution: 1:4000), PCNA (Cell Signaling Technology, Danvers, MA, USA; Dilution: 1:6000), Effluc (Promega, Madison, WI, USA; Dilution: 1:5000). The following secondary antibodies were used: HRP-conjugated anti-mouse (Cell Signaling Technology, Danvers, MA, USA; Dilution: 1:8000), HRP-conjugated anti-rabbit (Cell Signaling Technology; Dilution: 1:8000), HRP-conjugated anti-goat (Cell Signaling Technology; Dilution: 1:8000). The signals were detected using an electrochemiluminescence detection system (Thermo Fisher Scientific) according to the manufacturer’s protocol.

### Isolation of EVs

Cal62 and Cal62/Rluc cells were cultured with DMEM supplemented with 10% EV-depleted fetal bovine serum (filtered through a 0.22-µm syringe filter and then centrifuged for 18 h at 100,000 × *g* at 4 °C) for isolation of EVs. EVs were isolated as described previously^33^. Briefly, the supernatant was centrifuged at 300 × *g* for 10 min, 1500 × *g* for 20 min, and finally 2500 × *g* for 20 min (to remove cells and debris). The supernatant was filtered through a 0.45-µm syringe filter and centrifuged at 100,000 × *g* for 60 min. The pellet was resuspended and centrifuged at 100,000 × *g* for 60 min. The final pellet was resuspended in 50–100 µL of PBS and stored at −80 °C. All EVs were used within one week. All ultracentrifugation steps were performed (SW-28 rotor; Ultra-Clear tube) using the Optima^TM^ L-100 XP ultracentrifuge (Beckman Coulter, Brea, CA, USA). All centrifugation steps were conducted at 4 °C.

### Transmission Electron Microscopy (TEM)

EVs were imaged by TEM. Briefly, after isolation, EVs were fixed at 4 °C overnight. The fixative contained 2.5% glutaraldehyde in 0.01 M phosphate buffer at pH 7.4 (filtered through 0.22-µm filters) and was washed with PBS. EVs were post-fixed in 1% O_s_O_4_ (Taab Laboratories Equipment Ltd., Reading, UK) for 30 min. EV pellets were washed with distilled water and dehydrated with graded ethanol. EV pellets were negative-stained with 1% uranyl-acetate in 50% ethanol for 30 min and then embedded in Taab 812 (Taab), followed by polymerization at 60 °C overnight and ultra-sectioning for TEM. Ultra-thin sections were examined, and images captured with a HT-7700 transmission electron microscope (Hitachi, Tokyo, Japan) operated at 100 kV.

### Electrophoretic Light Scattering (ELS) Analysis

EV-Cal62/Rluc resuspended in PBS was further diluted by 200–400-fold in distilled water to determine the size and distribution with an ELS-Z (Otsuka Electronics, Osaka, Japan).

### Rluc Activity Assay for EV-Cal62/Rluc

EV samples were plated in white and clear-bottom 96-well plates with increasing concentrations of EV-Cal62 and EV-Cal62/Rluc (0, 25, and 50 μg) at the same volume. The substrate coelenterazine was added to each well. Rluc activity was determined by bioluminescence imaging as described above.

### Rluc Stability Assay

EV-CAL-62/Rluc or Free Rluc protein was incubated in 20% FBS in a PBS solution at 37 °C^33^. Stability of Rluc was evaluated by measuring Rluc activity at 0, 6, 12, 18, 24 hours for EV-CAL-62/Rluc. Stability of Free Rluc protein (Creative BioMart) was evaluated by measuring Rluc activity at 0, 1, 2, 3, 4 and 5 hours. The appropriate substrate coelenterazine was added to each well. Rluc activity was determined by BLI as described above.

### Rluc Stability Assay Recombinant Rluc Protein (r-Rluc)

Stability of Free Rluc protein (Creative BioMart) was evaluated by measuring Rluc activity at 0, 1, 2 and 3 hours. The appropriate substrate coelenterazine was added to each well. Rluc activity was determined by BLI as described above.

### Labeling EVs with DiD

To analyze the internalization of EVs, 20 µg of EVs were labeled with 1,1′-dioctadecyl-3,3,3′,3′-tetramethylindodicarbocyanine, 4-chlorobenzenesulfonate salt (DiD; Invitrogen), incubated for 20 min at room temperature, and washed with PBS. Next, a two-step OptiPrep density gradient ultracentrifugation was performed as described^9^.

### Immunostaining and Confocal Analysis of EV-CAL62 and EV-CAL62/Rluc

Immunofluorescence on EV-CAL62 and EV-CAL62/Rluc were performed as described^58^. DiD labeled and purified EV-CAL62/DiD and EV-CAL62/Rluc/DiD were added CAL62 cells for 2-hours, Fixed in 4% paraformaldehyde and permeabilized by 0.5% Triton-X-100. After a blocking step in 3% BSA in PBS, slides were stained overnight at 4 °C with anti-rabbit Rluc (Abcam; Dilution: 1:400) antibodies. After PBS washes and goat anti-rabbit FITC incubation (Abcam; Dilution: 1:500) were mounted with Vectastain anti-fade mounting medium with DAPI (Vector Laboratories, Burlingame, CA, USA). Images were acquired with a Zeiss super-resolution confocal microscope (LSM 5 Exciter, Zeiss, Oberkochen, Germany).

### Internalization Assay

The DiD-labeled EVs were incubated together with CAL62 parental cell lines for 1 h at 37 °C under 5% CO_2_ and were then fixed in methanol. Coverslips were then mounted using the VECTASHIELD anti-fade mounting medium (Vector Laboratories, Burlingame, CA, USA) and sealed. The cellular internalization of EVs was observed using an LSM 780 laser scanning microscope (Carl Zeiss). Total number of DiD (EV-CAL62/Rluc/DiD) positive cells were counted by two independent observers from CAL62 and CAL62/Effluc cells.

### Flow cytometry

Flow Cytometry Analysis was performed as described^59^, Briefly, The DiD-labeled EVs were incubated together with CAL62 or CAL62/Effluc cells for 1-hour at 37 °C under 5% CO_2_. Cells were washed with PBS (trice) and centrifuged for 3 min at 3500 g to remove free EVs. The supernatant was discarded, and the cells were resuspended in 1 mL of PBS for flow cytometry analysis on a BD Biosciences Aria III (BD Biosciences, San Jose, CA, USA).

### Animals

Five-week-old female BALB/c nude mice were purchased from Japan SLC, Inc. (Shizuoka, Japan). All procedures were reviewed and approved by Kyungpook National University (KNU-2012-43) Animal Care and Use Committee and performed in accordance with the Guiding Principles for the Care and Use of Laboratory Animals.

### Establishment of a CAL62/effluc Subcutaneous Animal Model

Nude mice were subcutaneously implanted with CAL62/effluc (5 × 10^6^ cells) on the right lower flank regions and allowed to grow for 6 weeks. The mice were anesthetized with 2.5% isoflurane (Merial, Lyon, France) and bioluminescent images were captured using the IVIS Lumina III imaging system after intraperitoneal injection of 100 µL of D-luciferin (3 mg/mouse; Caliper). BLI was subsequently assessed up to the 6^th^ week and mice were dived into two groups (EV-Cal62/Rluc or PBS).

### *In vivo* Imaging of EV-Cal62/Rluc Targeting Tumors

The mice were anesthetized with 2.5% isoflurane and bioluminescent images were captured using the IVIS III imaging system with an appropriate amount of coelenterazine administered intravenously after 5, 30, 60 and 120 min after EV-Cal62/Rluc (50 µg) (n = 3) injection to naïve mice, EV-Cal62/Rluc (50 µg) (n = 3) or PBS (n = 3) injection to tumor bearing mice through same route. Animals were sacrificed after 120 min and tumors were immediately excised and incubated with coelenterazine. Bioluminescent signals were captured and quantified as described above.

### Immunofluorescence (IF)

Tumor tissues were cryo-sectioned and processed for immunofluorescence assay as described previously^58^. CAL-62/Rluc- or PBS-injected mouse tumor sections were stained with Rluc (Abcam; Dilution: 1:400), followed by goat anti-rabbit FITC (Abcam; Dilution: 1:500); Effluc (Promega; Dilution: 1:300), and donkey anti-goat Alexa Fluor® 647 (Abcam; Dilution: 1:500). Tissue sections were mounted using VECTASHIELD mounting medium (Vector Laboratories, Burlingame, CA, USA). IF-stained sections were imaged under a confocal microscope (LSM 5 Exciter, Zeiss, Oberkochen, Germany).

### Statistical analysis

All data are expressed as the means ± standard deviation (SD). Two groups of data were statistically analyzed by *t*-test using GraphPad Prism7 software version 7.04 (GraphPad Software, Inc., La Jolla, CA, USA). A *P* value less than 0.05 was considered statistically significant.

## Electronic supplementary material

Supplementary information

## Data Availability

The authors declare that all the relevant data supporting the findings of this study are available within the article or from the corresponding author upon request.
