# Supplementary material for: New Optical Imaging Reporter-labeled Anaplastic Thyroid Cancer-Derived Extracellular Vesicles as a Platform for In Vivo Tumor Targeting in a Mouse Model
Source: Sci Rep. 2018 Sep 10;8:13509. doi: 10.1038/s41598-018-31998-y (PMC6131173; doi:10.1038/s41598-018-31998-y)
Supplement: Supplementary file 1 — Supplementary information [file 41598_2018_31998_MOESM1_ESM.pdf]

Supplementary information

New Optical Imaging Reporter-labeled Anaplastic Thyroid Cancer-Derived Extracellular Vesicles as a Platform for *In Vivo* Tumor Targeting in a Mouse Model

Prakash Gangadaran[1,2], Xiu Juan Li[1,2,3], Senthilkumar Kalimuthu[1,2], Oh Ji Min[1,2], Chae Moon Hong[1,2], Ramya Lakshmi Rajendran[1,2], Ho Won Lee[1,2], Liya Zhu[1,2], Se Hwan Baek[1,2], Shin Young Jeong[1], Sang-Woo Lee[1], Jaetae Lee[1] and Byeong-Cheol Ahn[1]*

[1]Department of Nuclear Medicine, School of Medicine, Kyungpook National University, Daegu, Republic of Korea. [2]Department of Nuclear Medicine, Kyungpook National University Hospital, Daegu, Republic of Korea. [3]Department of Radiology, Taian City Central Hospital, Taian, People's Republic of China.

*** Corresponding author**

Byeong-Cheol Ahn., M.D., Ph.D.,

Professor and Director,

Department of Nuclear Medicine,

School of Medicine, Kyungpook National University,

Kyungpook National University Hospital,

50, Samduk 2-ga, Jung Gu, Daegu-700-721, Republic of Korea.

Tel: 82-53-420-5583

Fax: 82-53-422-0864

Email: abc2000@knu.ac.kr

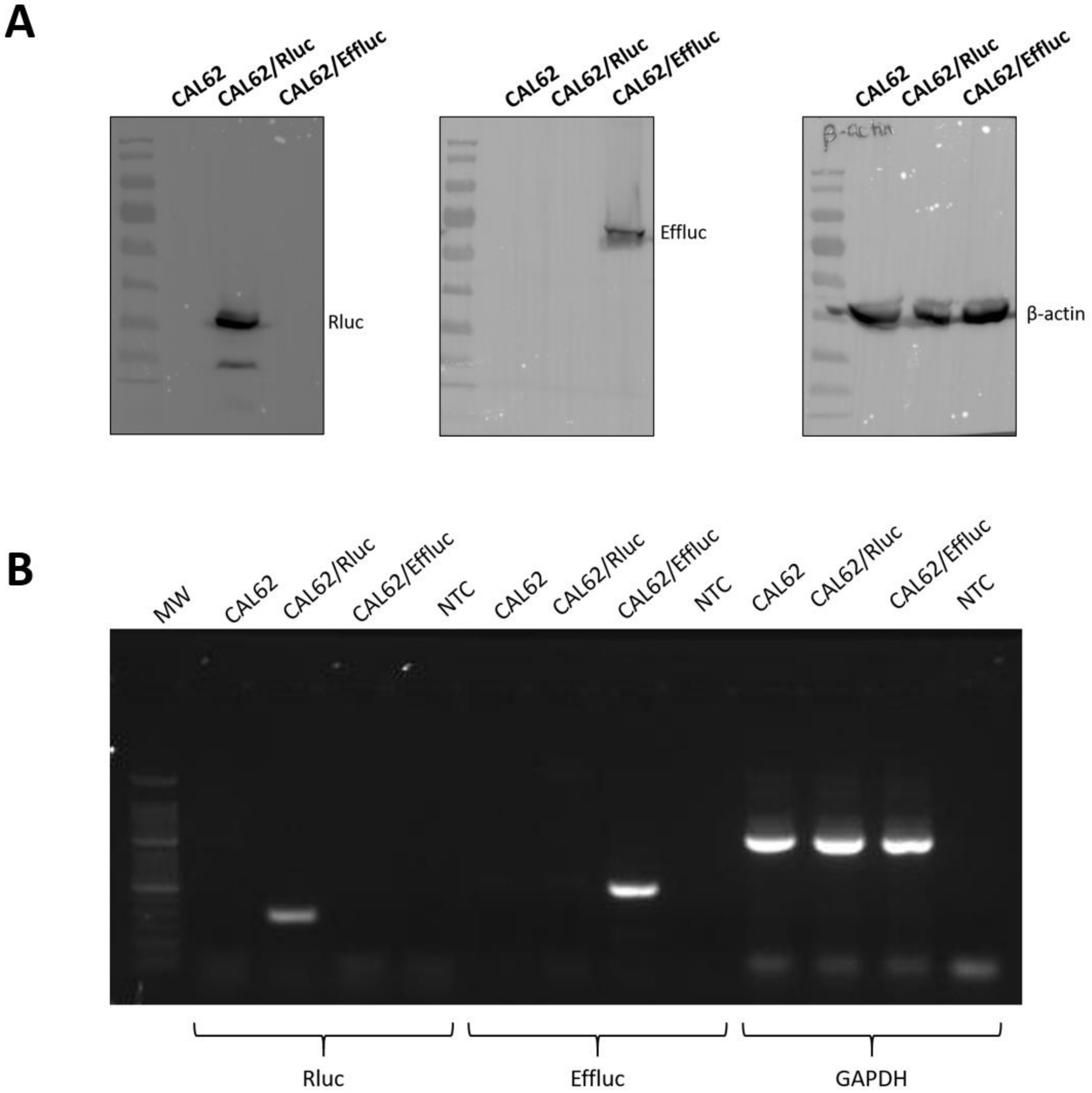

Supplementary Figure 1 : (A, B) Uncropped image of Western blot and RT-PCR analysis of figure 1E, F

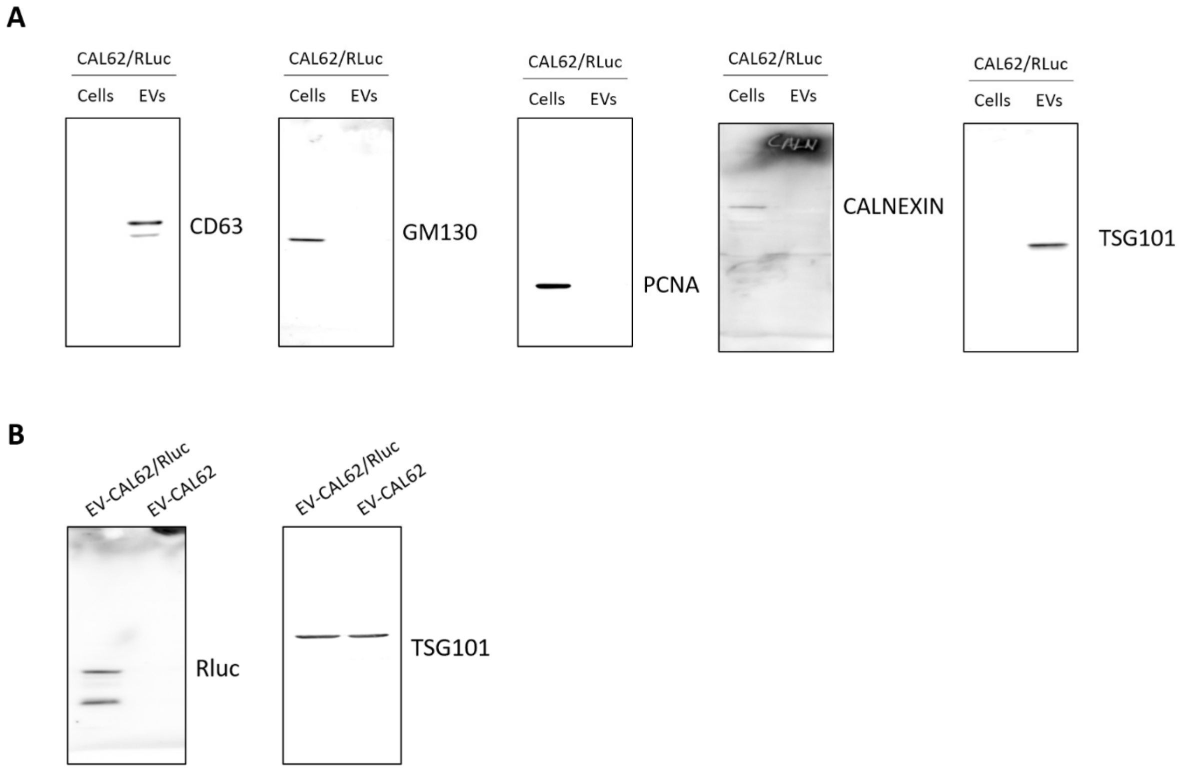

Supplementary Figure 2. (A, B) Uncropped image of Western blot analysis of figure 2B, 3C.

A

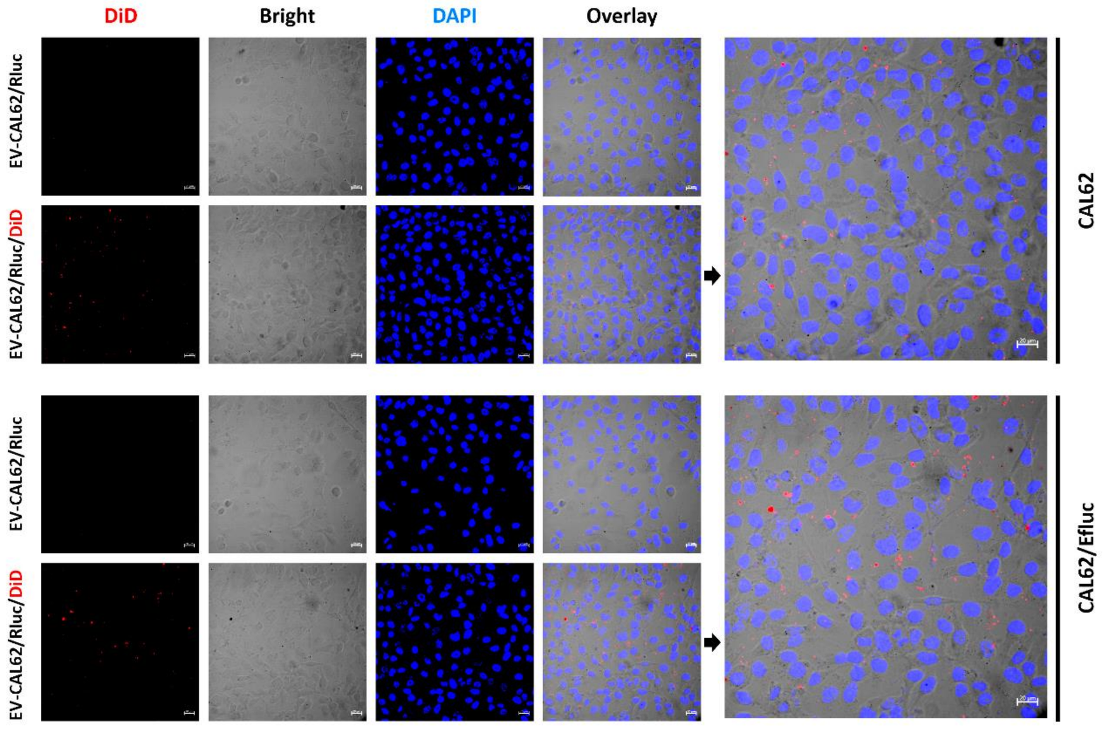

B

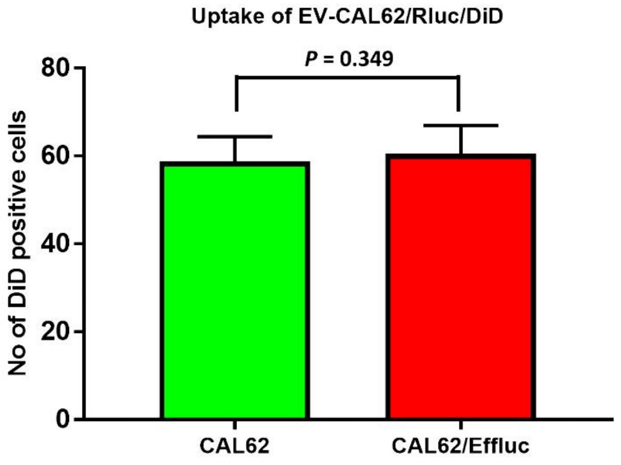

Supplementary Figure 3: Internalization of EVs into cancer cells. Confocal images of CAL62 or CAL62/Effluc cells incubated with unlabeled EV-CAL62/Rluc or DiD-labeled EV-CAL62/Rluc. Scale bar: 20 μm. **(B)** Quantification of DiD positive cells from confocal images of CAL62 or CAL62/Effluc treated with unlabeled EV-CAL62/Rluc or DiD-labeled EV-CAL62/Rluc was counted and represented in bar graph (p=0.349).
